# Next-Generation Sequencing Data and Clinical Features in Patients with Cleft Palate and Tooth Agenesis: A Systematic Literature Review

**DOI:** 10.3390/dj14080470

**Published:** 2026-08-02

**Authors:** Nisrine Boutahari, Lamiae Belayachi, Sonia Ghoul

**Affiliations:** 1 International Faculty of Dental Medicine, Health Sciences Research Center, College of Health Sciences, International University of Rabat, Technopolis Parc, Rocade of Rabat-Salé, Sala-Al Jadida 11100, Morocco; sonia.ghoul@uir.ac.ma; 2 International Faculty of Medicine, Health Sciences Research Center, College of Health Sciences, International University of Rabat, Technopolis Parc, Rocade of Rabat-Salé, Sala-Al Jadida 11100, Morocco; lamiae.belayachi@uir.ac.ma

**Keywords:** high-throughput nucleotide sequencing, anodontia, genes, craniofacial abnormalities, AXIN2 protein, human, mutation, congenital, hereditary, neonatal diseases and abnormalities

## Abstract

**Objectives**: The aims of this study were to explore the genetic variants identified by Next-Generation Sequencing (NGS) in patients presenting syndromic/non-syndromic cleft palate (CP) associated with tooth agenesis (TA) and to describe the observed phenotype–genotype correlations. **Methods**: A systematic review exploring PubMed, Scopus and Web of Science was conducted. Data extraction and bias assessment were performed. **Results**: From 227 screened articles, 8 studies were included. Second premolars were the most frequently missing teeth, followed by central incisors in non-syndromic CP cases. Genetic variants were most commonly reported in *IRF6*, *FGFR1*, *NOTCH2*, *CTNND1*, *ZFHX4* and *AXIN2*. Several mutations in these genes were associated with syndromic forms such as Pierre Robin Sequence, Kallmann syndrome, and Van der Woude syndrome. **Conclusions**: This study suggests a potential shared genetic pathway between CP and TA and supports further exploration of TA as a possible clinical indicator of syndromic cases. NGS emerges as a valuable exploratory tool for identifying such associations, though validation in larger patient cohorts remains necessary.

## 1. Introduction

Cleft palate (CP) is a congenital abnormality of the oral cavity characterized by an opening or split in the roof of the mouth that can impact both the hard and soft palates. It accounts for one-third of all orofacial clefts (OFCs) and affects approximately 1 to 25 per 10,000 newborns worldwide [1]. The incidence of CP is strongly impacted by ethnicity and race, with native Americans having the highest rates and Africans having the lowest [2]. Furthermore, females seem to be more susceptible to the defect than males (1:1.075) [2]. Anatomically, CP may be classified as complete, when the cleft extends through both the hard and soft palates, or incomplete CP when the cleft involves the soft palate alone or extends variably into the hard palate. According to the clinical features, CP can also be classified into two major groups [3]: (i) In isolated CP or non-syndromic CP (NSCP), clefting occurs as a single, isolated defect unassociated with any recognizable anomalies [4]. This form accounts for 54.8% of all CP cases. (ii) In syndromic CP, clefting is associated with additional anomalies affecting other organs as part of recognizable syndrome (27.2%) or unrecognizable syndrome (18%) [3]. The most prevalent syndromes with CP deformity include Apert Syndrome, associated with craniosynostosis; Pierre Robin Sequence (PRS), associated with underdeveloped jaw and breathing difficulty; Crouzon Syndrome, affecting the shape of the head and face; DiGeorge Syndrome, associated with heart defects; Loeys-Dietz Syndrome, associated with heart, bones, skin and organs defects; Treacher Collins Syndrome, affecting the growth of skull and facial bones; X-linked CP Syndrome with Ankyloglossia, affecting tongue development; and Bamforth–Lazarus Syndrome, associated with thyroid dysgenesis [3]. The multifactorial etiology of CP involves both unmodifiable factors (e.g., race/ethnicity, sex, and family history of clefts) and modifiable factors that act during the critical period spanning one month before to two months after conception [3]. In fact, during embryo development, maternal factors such as health/disease status, lifestyle, medication, and exposure to environmental teratogens can influence the intrauterine environment and the occurrence of CP. In addition to environmental factors, mutations in several developmental genes including Sonic hedgehog (Shh), bone morphogenetic protein (Bmp), fibroblast growth factor (Fgf), transforming growth factor beta (Tgf-β), and Wnt signaling contribute to CP development [5].

Additionally, patients with clefts often have dental anomalies, including tooth agenesis (TA). TA is frequently associated with unilateral palate patients, occurring in 48.8 to 75.9% in the cleft region and 27.2 to 48.8% in non-cleft areas. In the general population, the prevalence of TA ranges from 3.2 to 7.6% for men and 4.6 to 7.6% for women. It is suggested that the size of the cleft correlates with the number of missing teeth, with larger clefts leading to a higher incidence of agenesis [2]. The prevalence of TA in non-syndromic CP has been reported to range between 29.8% and 36.8%, predominantly affecting mandibular second premolars and maxillary lateral incisors, with mandibular agenesis occurring more frequently than maxillary agenesis [6]. Laterality is also suggested to influence dental patterns, as unilateral CP has been associated with a cleft-side predominance of maxillary TA, whereas bilateral CP tends to involve mandibular second premolars [2]. In terms of severity, complete forms of CP have been reported to show a higher involvement of mandibular TA compared with incomplete clefts. Syndromic CP has been associated with a higher prevalence of TA, reaching approximately 47.8%, frequently in association with PRS, and often displaying more extensive and symmetrical premolar involvement compared with non-syndromic cases [7].

According to the number of missing teeth, three types of TA are described: hypodontia (HD), oligodontia (OD), and anodontia (AD) [8]. HD is used to describe one to five missing teeth, whereas oligodontia OD is used for six or more missing teeth. AD is the most severe disease, with complete absence of tooth in both the deciduous and permanent dentitions [8]. TA can occur independently, resulting in a non-syndromic form of TA or in association with other dental defects and/or general medical conditions, leading to a syndromic form of TA [9]. Indeed, TA may have a common genetic etiology with CP, as revealed by numerous studies identifying more than 26 genes, including but not limited to *MSX1*, *PAX9*, *IRF6*, *TP63*, *BMP2*, *BMP4*, *WNT10A*, *WNT3*, and *AXIN2* [10]. For instance, the association of those genes with TA in CP patients has been established through various studies [8,11] that used different molecular biology and genetic analysis techniques, including Next-Generation Sequencing (NGS). Although TA is frequently observed in patients with CP, it is not present in all cases. This clinical heterogeneity raises questions about the underlying genetic differences between CP cases with and without associated dental agenesis. In this regard, a systematic review published by Boutahari et al. [12] on genes associated with non-syndromic TA detected by NGS revealed that various types of mutations ranging from novel mutations to missense, nonsense, duplication, deletion, and splicing mutations were identified by NGS in 19 genes. These included not only genes already known to be associated with TA (*MSX1*, *PAX9*, *EDA*, *EDAR*, *AXIN2*, *WNT10A*, *LAMA3*, *DKK1*, *COL17A1*, *IRF6*, *LRP6*, and *FGFR1*) but also new genes that had not been previously suspected (*CHD7*, *CREBBP*, *EVC*, *LEF1*, *ROR2*, *TBX22*, and *TP63*). In addition to that, some published studies reported that the *FGFR2*, *PAX9*, *TBX22*, *IRF6* and *MTR* genes could increase CP risk in patients without TA [5,13]. It is not clear whether different genes are expressed in patients with TA with non-syndromic CP and TA with syndromic CP. This highlights the need to further explore the genetic factors contributing to cases with both conditions. Thus, this study aims to identify and summarize genes associated with TA in patients with syndromic and non-syndromic CP as detected by NGS techniques and validated by Sanger sequencing to assess genotype–phenotype correlations.

## 2. Materials and Methods

The protocol for this systematic review was prospectively registered in PROSPERO on 10 July 2025 under registration number CRD420251037557.

### 2.1. Search Strategy

The literature search for the present systematic review and meta-analysis was conducted in accordance with the Preferred Reporting Items for Systematic Reviews and Meta-Analyses (PRISMA) guidelines (Table S1). A bibliographic search on the genes associated with TA in patients with CP detected by NGS was performed on 4th March 2025 and updated on 18th June 2026 using the electronic databases PubMed, Scopus, and Web of Science. The search strategy used 3 concepts. Each concept was developed as follows: (i) [((“Anodontia” [Mesh] OR “Tooth Agenesis” OR “Dental Agenesis” OR “Hypodontia” OR “Oligodontia”)] AND (ii) [“Cleft Palate” [Mesh]] AND (iii) [(“Next-Generation Sequencing” OR “High-Throughput Nucleotide Sequencing” [Mesh] OR “NGS” OR “Whole Exome Sequencing” OR “Whole Genome Sequencing” OR “Gene panel sequencing”))] (Table S2). These keywords were chosen based on the formulated research question developed using the SPiDER methodology. The problem statement using the SPiDER framework is reported in Table S3.

### 2.2. Eligibility Criteria

All the peer-reviewed articles published in English on genetic alterations detected in patients with TA and CP detected by NGS performing Whole-Exome Sequencing (WES), Whole-Genome Sequencing (WGS) and gene panel analysis were considered eligible for inclusion in this study. This study excluded narrative reviews and letters to the editors and included all randomized controlled trials, cross-sectionals, retrospective, cohort, case reports and case–control studies. Further exclusion criteria were: unavailable full text or abstract, studies with unavailable or insufficient data, studies where neither TA nor CP was mentioned, reports where species were not human and studies where genetic variants were not explored by NGS.

### 2.3. Study Selection

All records identified through the database searches were imported into Rayyan (Qatar Computing Research Institute) for reference management and screening. Duplicate records were identified and removed within the platform. Titles and abstracts were independently screened by two reviewers according to predefined inclusion and exclusion criteria. Full-text articles of potentially eligible studies were subsequently assessed using Rayyan. Any disagreements at either screening stage were resolved through discussion and consensus. When consensus could not be reached, a third reviewer was consulted.

### 2.4. Data Extraction and Quality Assessment

For each of the eligible studies, three types of data were collected independently by 2 reviewers through a data extraction grid: (i) publication data such as authors’ names and year of publication; (ii) clinical data described in the study such as number of involved patients, age, type of TA, relatedness, and missing-tooth type; and (iii) NGS data such as studied genes, methods and techniques of sequencing, coverage percentage, depth percentage, sample type, DNA quality evaluation, and Sanger validation. Detailed information regarding study design, sample source and control group number and characteristics are reported in Table S4. Risk-of-bias evaluation were assessed with “robvis”, a free-source web application. The selected assessment tool used was ROBINS-I, and the data were loaded manually. For each study, 5 domains of risk of bias were proposed: D1: phenotype misclassification bias; D2: genotyping errors; D3: insufficient data; D4: consanguinity; D5: confounding bias. A “high risk of bias” judgment in any domain puts the overall result at a high risk of bias. As to the interpretation of the risk-of-bias domains, a description of each variable is given in Table S5. For each domain, studies were rated low risk, some concerns, or high risk. A domain was rated “low risk” when sufficient methodological detail and appropriate procedures were reported, “some concerns” when information was incomplete or partially addressed, and “high risk” when clear methodological limitations or omissions were identified. The ratings of the risk of bias for all included studies were assessed by 2 reviewers independently. Discrepancies were resolved through discussion, and consensus was reached in all cases.

## 3. Results

### 3.1. Search Results

**Figure 1:**
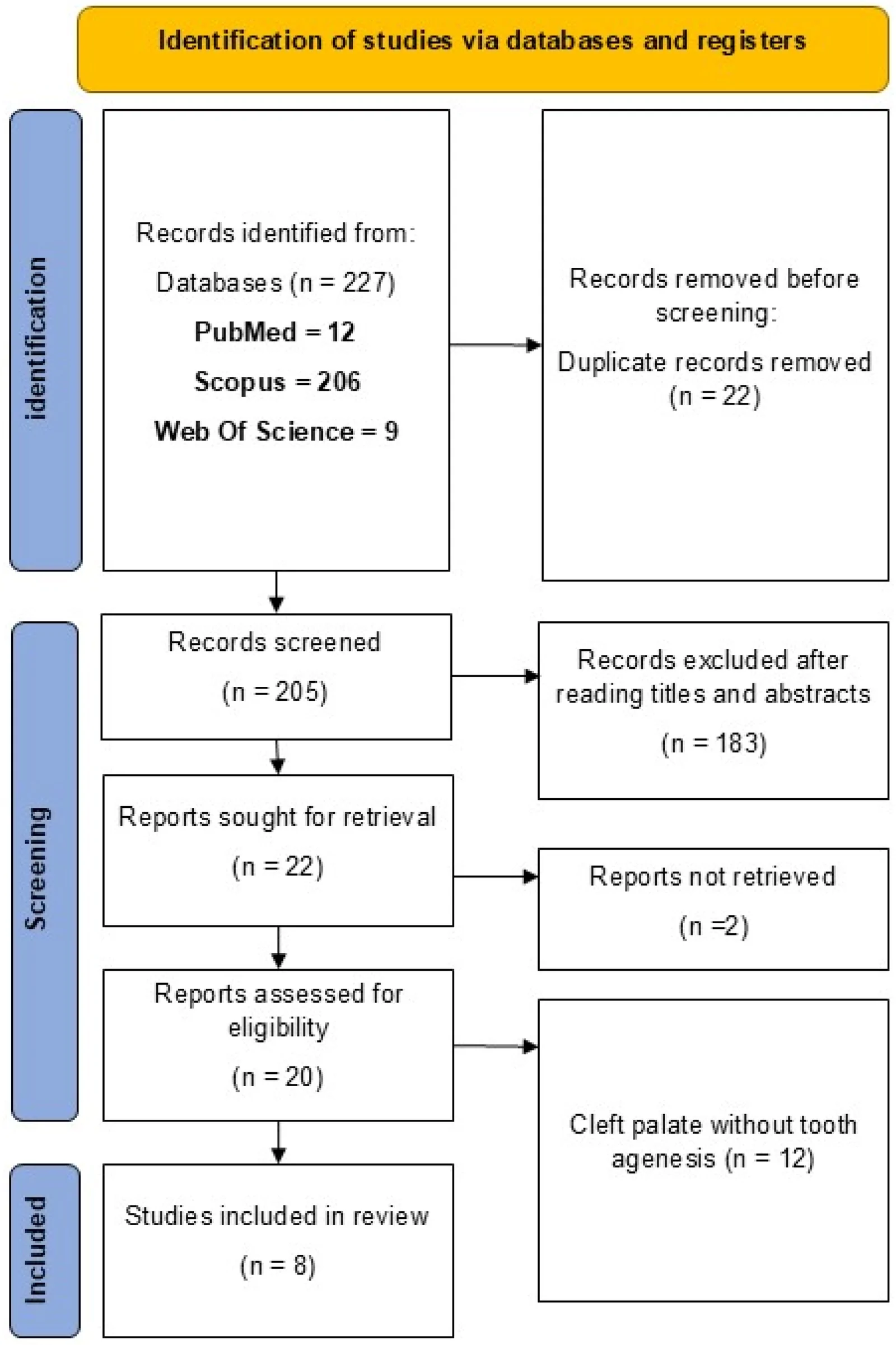
PRISMA flow diagram illustrating the identification, screening, and inclusion of studies reporting NGS findings in patients with CP and TA.

The initial literature search generated 227 articles. After removal of 22 duplicates, 205 articles were screened according to the title, abstract and study type. One hundred and twenty-three articles were excluded according to the predefined exclusion criteria. Twenty full-text articles were selected according to the predefined inclusion criteria. After full-text reading, eight articles were eligible to be included in the review as 12 papers studied CP without TA. The Preferred Reporting Items for Systematic Reviews and Meta-Analyses (PRISMA) diagram detailing the identification and selection process used in this study is shown in Figure 1.

### 3.2. Risk of Bias

**Figure 2:**
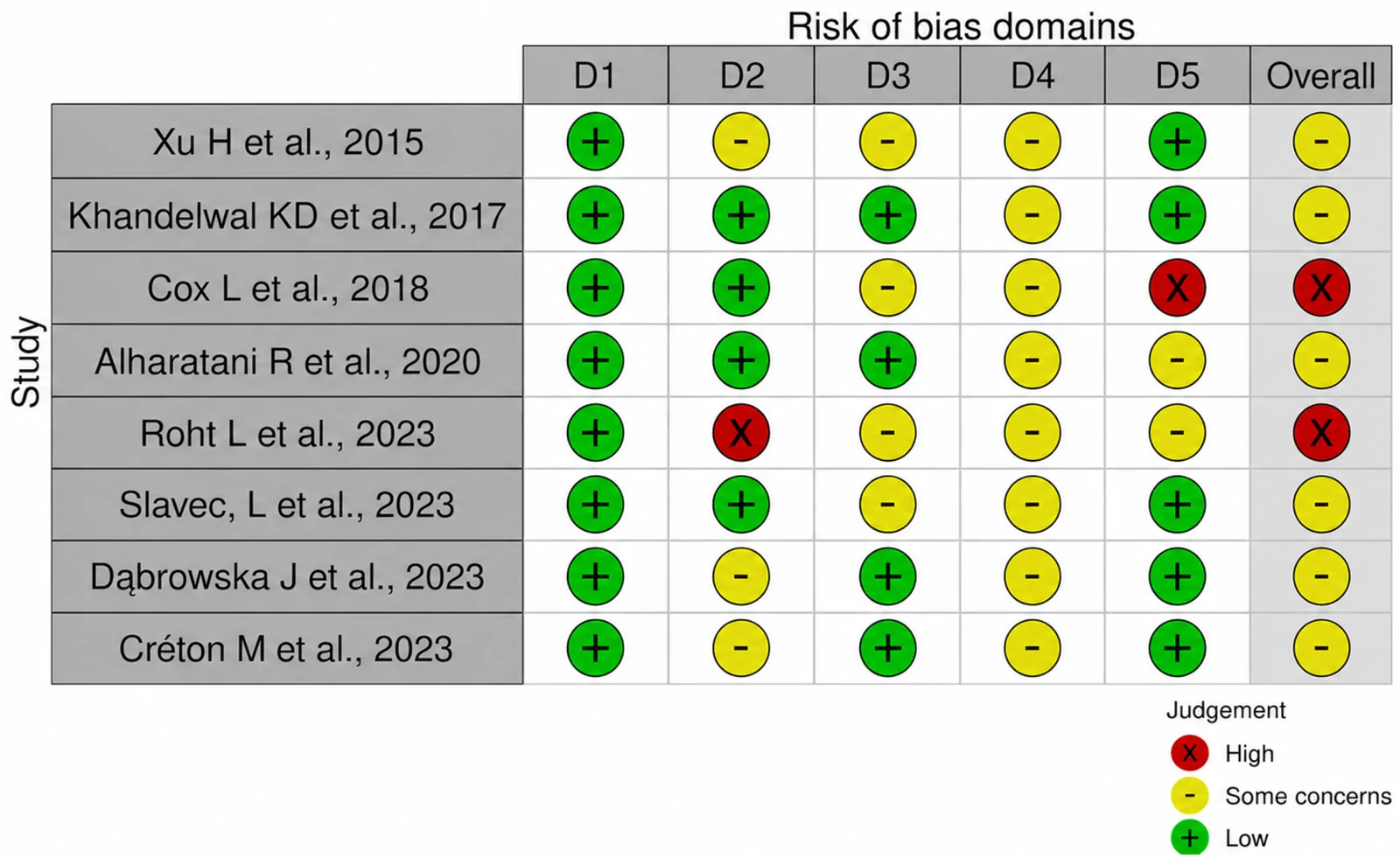
Evaluation of risk of bias of the included studies. D1: Phenotype misclassification bias. D2: Genotyping errors. D3: Insufficient data. D4: Consanguinity. D5: Confounding bias [14,15,16,17,18,19,20,21].

The quality and the assessment of the included studies were evaluated and are shown in Figure 2. Phenotyping errors showed low risk of bias in all studies. Meanwhile, insufficient data caused some concerns in four studies [14,15,16,17], cofounding bias caused some concerns in two studies [15,18], and high risk of bias was identified in one study [14]. Genotyping errors indicated high risk in one study [15] and some concerns in three studies [17,19,20], and consanguinity caused some concerns in all studies. Therefore, two study out of eight had a high overall risk of bias [14], and six studies presented some concerns [20,21].

### 3.3. Tooth Agenesis and Cleft Palate

**Figure 3:**
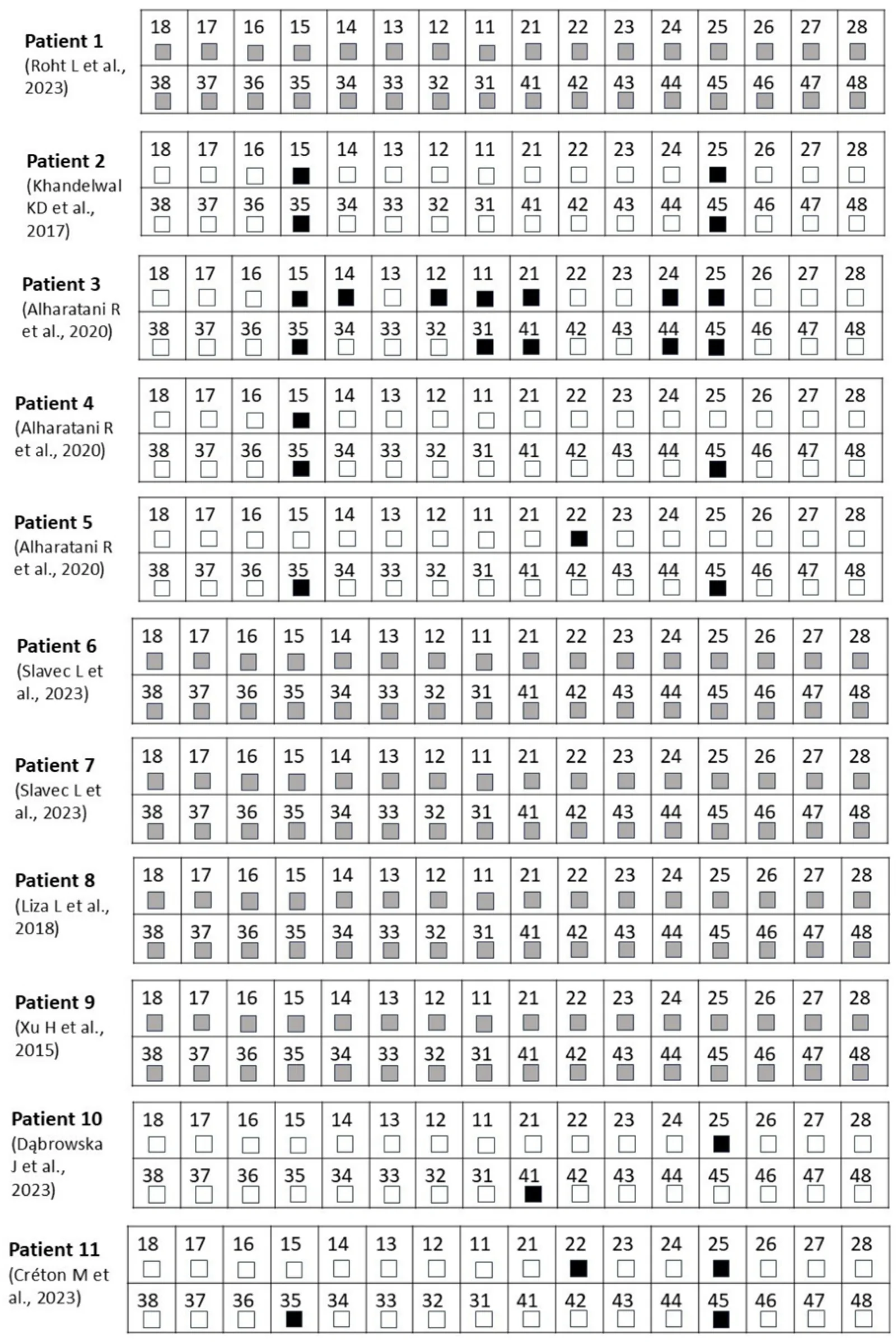
Patterns of maxillary and mandibular permanent TA in 11 patients with CP using the international tooth numbering system (FDI). Black boxes indicate congenitally missing permanent teeth; empty boxes indicate present teeth; Gray boxes indicate that tooth agenesis was reported but the tooth type was not specified in the original publication [14,15,16,17,18,19,20,21].

A total of 11 patients presenting with both CP and TA were identified. Ten cases (*n* = 10) were diagnosed with hypodontia [14,15,16,18,19,20,21], and only one was reported as “dental agenesis” without further specification [17]. The number of missing teeth varied across seven non-syndromic patients, ranging from 2 missing teeth to 12 missing permanent teeth (Table S6). Among these patients, six non-syndromic cases exhibited TA, most frequently affecting maxillary and mandibular second premolars, followed by maxillary and mandibular central incisors, maxillary and mandibular first premolars, and then maxillary lateral incisors. In one patient, the specific teeth affected by agenesis were not reported [14] (Figure 3). Several cases showed CP associated with additional features. Three syndromic associations among four patients were also reported, including Pierre Robin Sequence (PRS) [15], Van der Woude syndrome (VWS) with not only cleft palate but also bilateral cleft lip and lower lip pits [16], and Kallmann syndrome (KS) [17]. The TA pattern and the number of missing teeth were not reported in the syndromic cases.

### 3.4. NGS Approaches

WES was performed in four studies [14,16,18,19], while targeted gene panel sequencing was reported in three other cases [15,17,20]. One study used a targeted multiplex sequencing method with Molecular Inversion Probes (MIPs) [21]. An ion PGM sequencing platform was used in one study [17] and the Illumina Hiseq platforms Hiseq 4000 [18,20] and Hiseq 2500 [14] were used in three studies. The Illumina Novaseq 6000 was used in one study [16], and the Illumina NextSeq500 was used in another study [21], but it was not specified which sequencing platform was used in one study [19]. Sequencing coverages of 92% [17] and 98.5% [20] were described in two studies and were undetermined in the other six studies. Sequencing depths were specified as 50 times by Alharatani [18] and Roht [15], greater than 107 times in the article by Slavec [16], greater than 500 times in that by Khandelwal [21], and 1402 times by Xu [17] and were not specified in the articles in [14,19,20] (Table 1). Sanger sequencing was used in all included studies to validate the identified variants [14,15,16,17,18,20,21].

### 3.5. Phenotype–Genotype Associations

**Figure 4:**
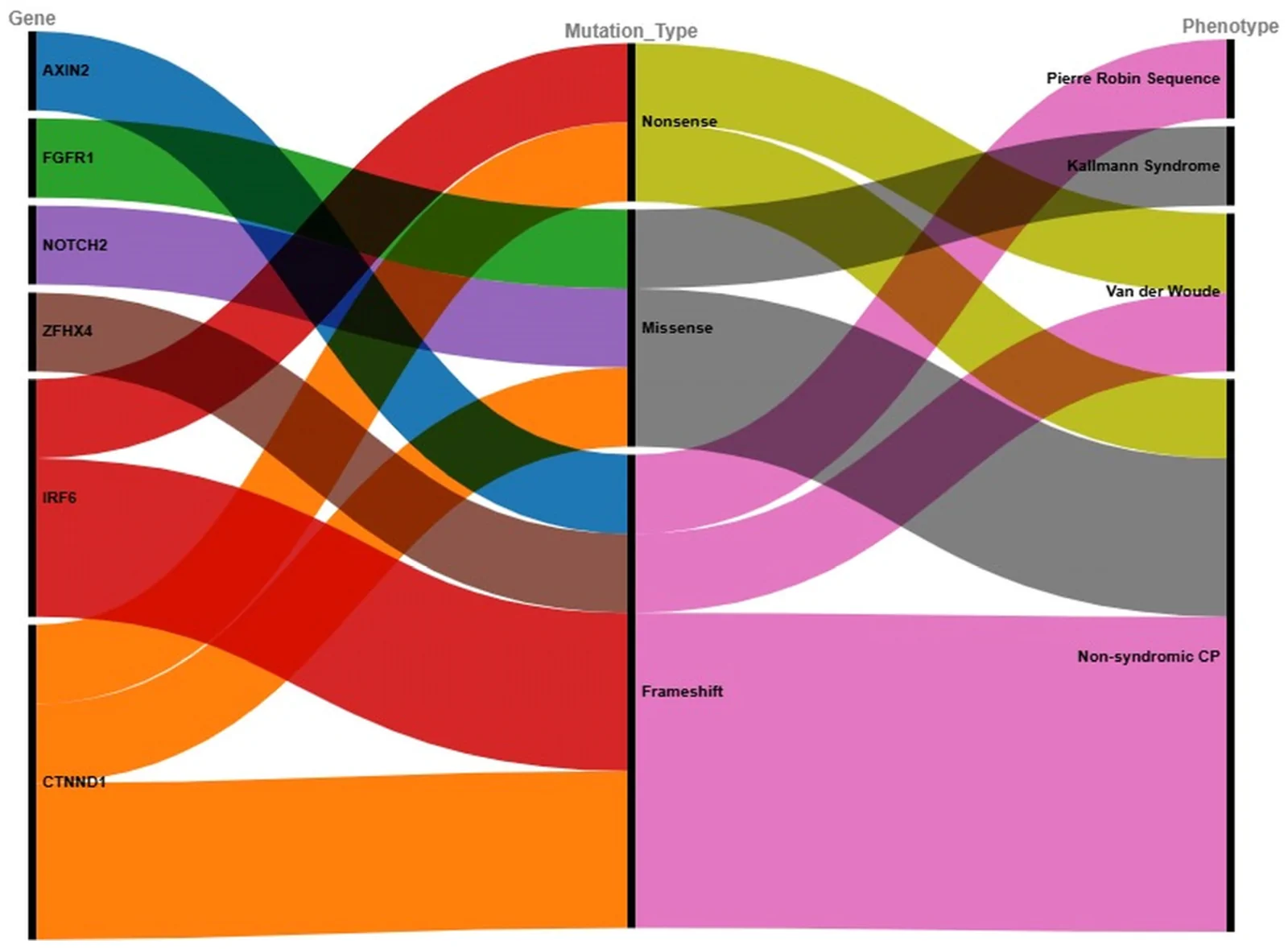
Alluvial diagram showing the relationships between genes, variant types (missense, nonsense, frameshift, and splicing), and the syndrome status of CP.

Additional analysis of NGS data revealed distinct genotype–phenotype relationships in patients with CP and TA, as summarized in Figure 4. The alluvial diagram demonstrates that specific mutations in six key genes (*FGFR1*, *AXIN2*, *NOTCH2*, *IRF6*, *ZFHX4* and *CTNND1*) were associated with distinct clinical presentations. *FGFR1* missense mutations aligned with KS, *AXIN2* frameshift variants corresponded to PRS, and *NOTCH2* missense mutations occurred in non-syndromic CP. *IRF6* nonsense and one frameshift mutations associated with VWS, while a second *IRF6* frameshift appeared in non-syndromic cases. *CTNND1* displayed all three mutation types (nonsense, missense, and frameshift) exclusively in non-syndromic CP and TA. The *ZFHX4* frameshift mutation was not associated with any syndrome.

### 3.6. Gene Mapping

**Figure 5:**
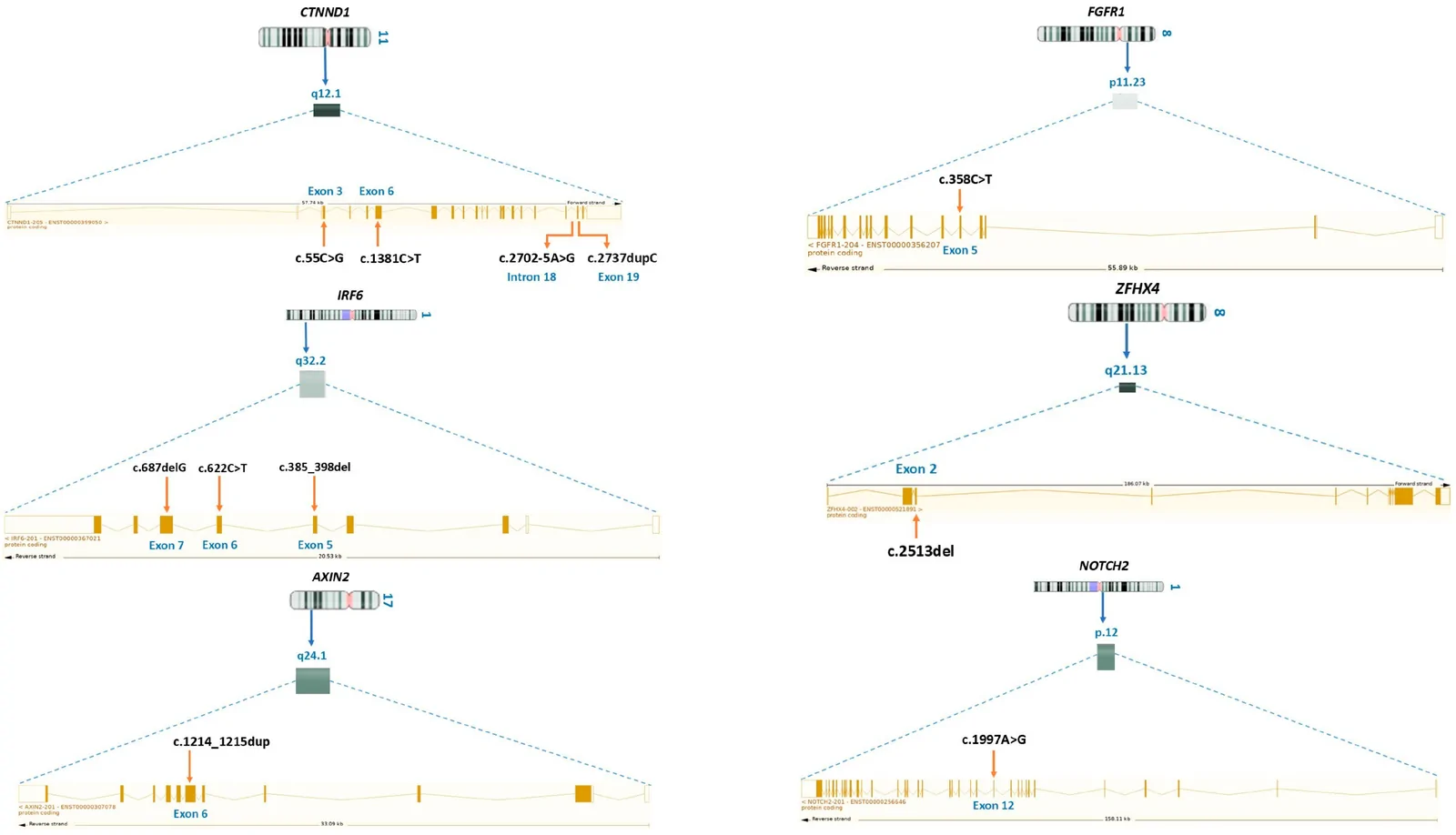
Chromosomal mapping of the 6 key genes identified in 11 patients with CP and TA (*IRF6*, *AXIN2*, *CTNND1*, *FGFR1*, *ZFHX4* and *NOTCH2*). Each gene is shown with its chromosomal location and locus, illustrating the genomic distribution of several variants.

Six distinct genes were identified with specific mutations that may be associated with TA in syndromic and non-syndromic patients with CP. These variants were located across different chromosomes and loci, involving various mutation types, and affecting different regions of chromosomes (Figure 5). A missense variant, c.358C > T, was detected in *FGFR1* on chromosome 8p11.23, exon 5, reverse strand. A frameshift variant, c.1214_1215dup, was identified in *AXIN2* on chromosome 17q24.1, exon 6, reverse strand. Four variants were found in *CTNND1* on chromosome 11q12.1: (i) a missense mutation, c.55C > G, on exon 3; (ii) a nonsense mutation, c.1381C > T, on exon 6; (iii) and two frameshift mutations, c.2737dupC and c.2702-5A > G, on exon 19 and intron 18, respectively, all located on the forward strand. On chromosome 1, two genes were affected. In *NOTCH2* (p12), a missense mutation, c.1997A > G, was found on exon 12, reverse strand. In *IRF6* (q32.2), a nonsense mutation, c.622C > T, and two frameshift mutations, c.687delG and c.385_398del, were identified respectively on exons 6, 7 and 5, all on the reverse strand. A frameshift variant, c.2513del, was detected on exon 2 of *ZFHX4*, on chromosome 8 (Table S7).

## 4. Discussion

This study explored the genetic basis of TA in patients with syndromic and non-syndromic CP using NGS through a systematic review of the literature. Our search strategy focused on three databases (Pubmed, Scopus and Web of Science) and applied the SPiDER methodology to formulate the research question as recommended for qualitative systematic reviews [22]. Across seven eligible studies comprising eleven patients, six potential genes (*IRF6*, *AXIN2*, *CTNND1*, *FGFR1*, *ZFHX4* and *NOTCH2*) were identified in both syndromic and non-syndromic CP cases associated with TA. In terms of dental patterns, this study reported that mandibular and maxillary second premolars were the most commonly missing teeth followed by maxillary and mandibular central incisors.

Concerning the data quality, two papers with low quality [14,15] due to potential population stratification within the analyzed cohort and genotyping errors were identified. In fact, such factor may result in false-positive or false-negative variant identification and lead to spurious associations between the genotype–phenotype associations when allele frequencies differ across ethnic backgrounds rather than being related to disease status. Nevertheless, this paper was retained because it included an independent replication cohort which strengthens the validity of the findings and reduces the likelihood of false positive mutations. Thus, eight papers were considered in our study. Overall, while six studies showed moderate risk of bias, due to incomplete phenotypic reporting (D3), consanguinity status, and potential population stratification (D5), genotyping reliability was consistently high across four studies due to adequate report of NGS quality metrics in these studies. These factors support moderate confidence in gene identification while limiting the strength of genotype–phenotype correlations, thus highlighting the need for precise and complete phenotyping descriptions and appropriate control of population stratification in future studies.

Concerning the NGS approaches, our study demonstrated that MIP sequencing and gene panel sequencing had higher sequencing depth than WES. These results align with those described in another study [23]. Gene panels and MIP sequencing demonstrate superior sensitivity in targeted regions, achieving coverage rates up to 98.8% at 20× depth for panels in neuromuscular genes [24] and 97.3% at >30× for MIPs across sodium channel genes [25], whereas WES typically achieved 93–96% coverage at 20× due to uneven capture efficiency and off-target reads [24]. Such technical differences may influence variant interpretation, as WES may fail to detect variants in regions with low read depth, increasing the risk of false-negative results. In contrast, targeted gene panels and MIP-based approaches generally achieve higher and sequencing depth across selected genes, increasing sensitivity for variant detection within these regions. Consequently, the absence of a variant in targeted sequencing data is more likely to reflect a true negative for the covered genes, whereas absence in WES data may reflect insufficient coverage rather than true variant absence. Despite its limitations, WES remains advantageous for identifying rare or novel variants in genes not previously associated with the phenotype and is particularly useful for genetically heterogeneous conditions such as TA [23].

Concerning TA and CP, our study involved 11 patients with 30 missing teeth. Second premolars were reported as the most commonly missing teeth, followed by central incisors. This finding confirmed previous published studies revealing that hypodontia of second premolars is often present when agenesis occurs outside the cleft region and hypodontia of lateral incisors is more common in the cleft area [26,27,28], hypothesizing that the absence of second premolars may be driven by distinct genetic and developmental factors rather than direct cleft interference. Moreover, it is astonishing that canines remain present in all patients with CP, while their agenesis was commonly described in non-syndromic TA in the absence of CP [12]. This discrepancy suggests that TA in patients with CP may be caused by both the cleft itself and other genetic factors. Considering gene expression patterns and regulatory mechanisms behind the specific development of each tooth, it is suggested that the genes involved in CP are also expressed during the development of second premolars, notably homeobox genes such as *MSX1* and *PAX9* [29]. Furthermore, it has been reported that syndromic CP involving syndromes like PRS, VDW and KS is more frequent than non-syndromic CP [30]. With the development in molecular technology, extensive research has followed to identify the underlying genes and variants associated with these syndromes; for example, 70% of VWS cases are found to be due loss-of-function variants in Interferon Regulatory Factor 6 (*IRF6*) [31]. While our *IRF6* variants disrupt protein function, prior studies implicate non-coding regions like MCS-9.7 in altering the binding site of *IRF6* within this enhancer, which was proven to predispose individuals to CP in both their syndromic and non-syndromic forms [32]. Moreover, published studies reported that palates in mutant embryos lacking *IRF6* failed to fuse; however, *IRF6* overexpression restored this process [33]. *AXIN2*, a key regulator of the WNT pathway, is essential to controlling cell fate during craniofacial morphogenesis and has been preserved throughout evolution [34]. Mutations in *AXIN2* have also been linked to developmental abnormalities such as TA and OFCs, as well as cancers like colon cancer, brain cancer and leukemia, among others [34,35]. A published study by Letra et al. found that during development, AXIN2 and IRF6 proteins colocalize in the tissues of the secondary palate, nose, mouth, and eye epithelium [34]; however, their simultaneous expression in these relevant tissues does not necessarily imply that these molecules interact during their intended roles and need further investigation. Located in 8p11.23, *FGFR1* is considered a key gene for KS. Mutations on this gene are also described as causative for other syndromes, with some of them including OFCs and dental anomalies [8], like a gain-of-function *FGFR1* mutation associated with KS and loss-of-function mutations in craniosynostosis presenting OFCs. Relevant insights into a possible common *FGFR1*-related mechanism that may contribute to the dual etiology of OFCs and TA have been reported by Phan et al. [8]. *CTNND1* encodes catenin delta-1 (also known as p120ctn), an Armadillo repeat protein that interacts closely with E-cadherin, and according to a published study, mutations located on exons 6, 7 and 14 of *CTNND1* are predicted to affect transcript isoforms 1 and 3 and probably lead to nonsense-mediated RNA decay, leading to haploinsufficiency [36]. It was also recently identified as the cause of blepharocheilodontic syndrome, of which CL/P is a variable feature [14]. In the same study by Cox et al. [14], it was showed that the knockout of *CTNND1* in mice supports a causative role in non-syndromic CL/P pathogenesis. The involvement of *CTNND1* is supported by the diversity in variant type (nonsense, frameshift, splicing, and missense) as well as the clustering of missense variants within the protein [14]. *ZFHX4*, a homeobox gene, has been widely reported to be linked to both orofacial clefting and tooth agenesis. A published study by Ishorst et al. detected a heterozygous 86 kb de novo deletion affecting exons 4–11 of *ZFHX4* in non-syndromic CL/P [37]. Genetic and phenotypic data from the same study demonstrated that *ZFHX4* variants can lead to both non-syndromic and syndromic forms not only of CL/P but also of CPO. Expression analysis in single-cell RNA-sequencing data from mouse embryos and zebrafish larvae at relevant time-points supported an important role of *ZFHX4* in craniofacial development. Additionally, CRISPR F0 knockout of *ZFHX4* and morpholino knockdown in zebrafish showed an underdeveloped and abnormally shaped ethmoid plate and cartilaginous jaw resembling micrognathia. Another study reported that loss of function resulted in distinct craniofacial defects, such as cleft palates in mice and ethmoid plate deformation in zebrafish [38]. Moreover, findings from a previous study indicate an important role of *ZFHX4* in craniofacial and brain development through interaction with proteins involved in chromatin organization, as its loss of function also caused microdeletion syndrome [39]. Notably, some of the genes identified in this review, particularly *AXIN2*, *IRF6* and *FGFR1* play essential roles not only in palatal development but also in odontogenesis [40]. This overlap supports the hypothesis that molecular disturbances contributing to CP formation may simultaneously impair tooth development. Such a mechanism could partially explain the frequent co-occurrence of TA in patients with CP, especially when no direct environmental factors are identified.

### Limitations

One of the main limitations of this systematic review is the limited number of included studies, which may restrict the generalizability of the findings. The heterogeneity in study design, sequencing approaches, clinical findings across the included articles complicates direct comparison of results. Moreover, functional validation of the identified variants was often lacking, making it difficult to confirm their causal role in TA and CP. Another limitation of this systematic review is the frequent lack of specification regarding CP laterality, severity, and detailed tooth-specific agenesis patterns, which restricted the assessment of how CP subtypes influence the prevalence and distribution of TA. Additionally, the lack of detailed phenotypic characterization, together with limited control for population stratification, represents an important limitation that restricts robust genotype–phenotype interpretation in the included studies. Furthermore, future studies should also consider stratifying analyses by sequencing methodology to account for these differential sensitivity profiles and avoid conflating datasets with inherently different false-negative rates.

## 5. Conclusions

This systematic review identified six key genes (*IRF6*, *AXIN2*, *CTNND1*, *FGFR1*, *ZFHX4* and *NOTCH2*) on chromosomes 1, 8, 11 and 17 that may be involved in the genetic etiology of TA in patients with CP, based on available NGS findings. These genes were associated with both syndromic and non-syndromic forms, with various mutation types suggesting a spectrum of phenotypic severity. In syndromic cases, it is hypothesized that TA may represent an early and clinically accessible indicator of underlying genetic disorders. Additionally, this study revealed that mandibular and maxillary second premolars were the most commonly missing teeth outside the cleft region, while maxillary central incisors were the most frequent absent tooth in the cleft region. Although only seven studies met the inclusion criteria, their findings suggest a potential genetic overlap between CP and TA, and support the role of NGS as an exploratory tool for investigating genotype–phenotype correlations. Larger, well-characterized cohorts are also required to validate these preliminary observations and clarify their diagnostic and clinical relevance.

## Figures and Tables

**Table 1:** The different technical approaches of NGS employed for analyzing various types of cleft palate and tooth agenesis.

Sequencing Method	Sequencing Platform	Sequencing Depth	Sequencing Coverage	Type of Cleft Palate and Tooth Agenesis	References
Gene panel	Illumina	≥50×	-	Syndromic	Roht L. et al., 2023 [15]
Targeted multiplex sequencing (MIP)	Illumina NextSeq500	≥500×	-	Non-syndromic	Khandelwal K.D. et al., 2017 [21]
Whole-Exome Sequencing	Illumina Hiseq	50×	-	Non-syndromic	Alharatani R. et al., 2020 [18]
Whole-Exome Sequencing	Illumina Novaseq 6000	>107×	-	Syndromic	Slavec L. el., 2023 [16]
Whole-Exome Sequencing	Illumina Hiseq 2500	-	-	Non-syndromic	Cox L. et al., 2018 [14]
Gene panel	Ion PGM	1402×	92%	Syndromic	Xu H. et al., 2015 [17]
Gene panel	Illumina Hiseq 4000	-	98.5%	Non-syndromic	Dąbrowska J. et al., 2023 [20]
Whole-Exome Sequencing	-	-	-	Non-syndromic	Créton M. et al., 2023 [19]

## Data Availability

The original contributions presented in this study are included in the article/Supplementary Materials. Further inquiries can be directed to the corresponding author.

## Supplementary Materials

The following supporting information can be downloaded at: https://www.mdpi.com/article/10.3390/dj14080470/s1, Table S1: PRISMA 2020 checklist [41]; Table S2: Complete strings for each database explored in the review with the exact search dates; Table S3: Problem statement using the SPiDER framework; Table S4: Study Design, Sample Source, and Control Groups for Included Studies; Table S5: Definition of the 5 risks of bias domains; Table S6: Clinical characteristics of patients with CP and TA Tooth agenesis patterns and syndromic status in patients with CP; Table S7: Summary of genes, variant types, inheritance patterns, and phenotypes in patients with CP and TA.
